# Repositioning health education as a core nursing function: a commentary

**DOI:** 10.1177/17449871251358627

**Published:** 2025-08-23

**Authors:** Sarah Bekaert

**Affiliations:** Faculty of Humanities and Social Sciences, Oxford Brookes University, Oxford, UK

Dorothea Orem’s Self-Care Deficit Nursing Theory (1985) is a foundational grand theory that emphasises health education as a key nursing role, positioning nurses as facilitators who support individuals in meeting their self-care needs. In Orem’s framework, self-care requisites refer to the essential needs individuals need to meet to maintain their health. These fall into three categories: universal requisites which include basic needs such as breathing, hydration, nutrition, hygiene, rest, safety, social interaction and normal development; developmental requisites that relate to specific life stages or transitions (e.g. adolescence, pregnancy, ageing) and health-deviation requisites that occur in cases of illness or injury and may include taking medications, managing symptoms, or adapting to altered physical function. In addition, a person’s ability to meet these needs, or their self-care agency, depends on factors such as age, knowledge, physical and emotional capacity, motivation, culture, environment, and access to resources.

The study by Dawood et al. (2025) exploring the positive impact of a nurse-led education module for patients fitted with a double-J (DJ) stent in urology highlights the relevance of Orem’s theory in practice. In this quasi-experimental study, 50 patients were split into a control group (receiving usual support) and an intervention group (receiving a structured nursing educational module). Patients’ knowledge about ureteral stones and DJ stents, how to manage them, how to monitor for signs of infection, and considerations around life-style aspects, as well as discomfort levels, were measured. Prior to the intervention, knowledge was low in both groups. However, one month after implementing the instructional module, only the intervention group achieved maximum knowledge scores. Education improved patients’ ability to monitor for infection, maintain mobility, and adopt healthy post-procedure behaviours, thereby reducing complications and further healthcare needs. The intervention group also experienced less pain and discomfort.

When individuals are unable to meet demands incurred by illness, Orem states that a self-care deficit occurs, necessitating nursing intervention. Nurses then step in to support, compensate, or educate patients, with the goal of restoring or developing their ability to care for themselves. Orem’s theory empowers nurses to educate, support, and gradually transfer responsibility from full dependence to independent self-management. The consequent positive outcomes for the patient is adeptly evidenced in the clear benefits for those who received the nurse-led education in Dawood et al.’s (2025) study. Furthermore, systematic review confirms that designing effective educational interventions improve patient outcomes, promotes treatment adherence, prevents complications and readmissions, and empowers patients, while also contributing to system efficiency and cost savings (Wang et al., 2024).

Despite its importance, the health education role of nurses is consistently marginalised. Research highlights a shift in nursing priorities towards medical and administrative tasks, often at the expense of patient education (Alrowili et al., 2024). Due to workforce shortages and rising workloads, nurses report that patient education is often compromised (Linnaviori et al., 2024). Dawood et al.’s (2025) study contributes to the growing body of evidence that health education should be an integral part of nursing care plans, supporting a renewed focus on education as a core nursing function.

Helpful theory to assist nurses in this role include the Health Belief Model (Rosenstock, 1974) and Prochaska and DiClemente’s (1983) Stages of Change Model. The Health Belief Model offers a valuable framework for structuring nursing education. It proposes that individuals are more likely to adopt healthy behaviours if they believe they are at risk (perceived susceptibility), recognise the seriousness of the condition (perceived severity), believe in the benefits of taking action, and feel confident they can overcome barriers. In nursing, the Health Belief Model supports a patient-centred approach to education. In the DJ stent study (Dawood et al., 2025), nurses tailored education by first eliciting patients’ existing knowledge and then adapting the module content accordingly. This approach reflects the Health Belief Model, allowing nurses to address patient fears, correct misconceptions, highlight benefits and boost confidence, making the education more relevant and empowering.

To enhance such person-centredness even further, nurses should also consider integrating the Stages of Change Model (Prochaska & DiClemente, 1983) into planned health education. This model views behaviour change as a process that includes: precontemplation, contemplation, preparation, action, maintenance, and termination (with relapse seen as a natural part of the cycle). Patients may not always be ready for change (in the precontemplation stage) or need to revisit key information for maintenance, or after relapse. This model articulates the need for nurses to assess a patient’s readiness to change and tailor education accordingly. To provide ‘stage-specific’ support, this may range from awareness-raising, to practical skill teaching, to employing strategies to boost self-efficacy using goal-setting, to demonstration and encouragement, and to normalise and manage relapse - helping patients re-engage with behaviour change. By aligning educational strategies with a patient’s stage of readiness, nurses can ensure that interventions are timely, respectful and effective – moving beyond didactic teaching to truly transformative care.
